# *APOE* genotype and biological sex regulate astroglial interactions with amyloid plaques in Alzheimer’s disease mice

**DOI:** 10.1186/s12974-022-02650-4

**Published:** 2022-12-01

**Authors:** T. L. Stephen, B. Breningstall, S. Suresh, C. J. McGill, C. J. Pike

**Affiliations:** Leonard Davis School of Gerontology, University of Southern California, 3715 McClintock Avenue, Los Angeles, CA 90089-0191 USA

**Keywords:** Alzheimer’s disease, Apolipoprotein E, Astrocytes, Glial interactions, Sex

## Abstract

The most significant genetic risk factor for developing late-onset Alzheimer’s disease (AD) is the ε4 allele of apolipoprotein E (*APOE4*). *APOE* genotype and biological sex are key modulators of microglial and astroglial function, which exert multiple effects on AD pathogenesis. Here, we show astroglial interactions with amyloid plaques in the EFAD transgenic mouse model of AD. Using confocal microscopy, we observed significantly lower levels of astrocytic plaque coverage and plaque compaction (beneficial effects of glial barrier formation) with *APOE4* genotype and female sex. Conversely, neurite damage and astrocyte activation in the plaque environment were significantly higher in *APOE4* carriers and female mice. Astrocyte coverage of plaques was highest in *APOE3* males and poorest in *APOE4* females. Collectively, our findings provide new insights into the roles of astroglia and highlight the importance of addressing independent and interactive effects of *APOE* genotype and biological sex in understanding processes contributing to AD pathogenesis.

**Supplementary Information:**

The online version contains supplementary material available at 10.1186/s12974-022-02650-4.

## Introduction

Alzheimer’s disease (AD) is an age-related neurodegenerative disease and the most common cause of dementia. A primary pathological hallmark of AD is the accumulation of β-amyloid protein (Aβ), which is implicated in driving neurodegenerative cascades [1], in the form of extracellular plaques. Another component of AD pathophysiology is activated glia. Both microglia and astrocytes exhibit activated phenotypes and are often co-localized with plaques [2]. While microglia are known to exhibit both positive and negative outcomes in AD, much less is known about the role of astrocytes, even though their presence near plaques is well-documented [3].

Astrocytes are the most numerous cell type in the brain and display extensive heterogeneity and phenotypic plasticity [4, 5]. Their role ranges from scar formation to energy provision and essential synapse maintenance [6]. These responses are altered and can be severely compromised when astrocytes adopt an activated phenotype [7, 8]. However, it is unclear whether reactive astrocytes closely associated with amyloid plaques play a protective and or harmful role in regulating AD. One way in which glia modulate AD pathogenesis is via interactions with amyloid plaques. For example, microglial processes form a barrier-like network around plaques that is associated with restricted outward expansion of Aβ plaque fibrils and protection against neuritic damage [9]. Whether astrocytes form similar protective barriers is not known.

Development of AD is significantly affected by several risk factors. The most significant genetic risk factor for AD is the ε4 allele of apolipoprotein E (*APOE4*), which increases risk up to 15-fold compared to the more prevalent ε3 allele (*APOE3*) [10]. ApoE is an abundant glycoprotein that is synthesized and secreted in the CNS mainly by astrocytes [11] and regulates several AD-related processes, including Aβ uptake and degradation [12, 13], that are mediated in part by astrocytes [11]. A complex interplay exists between sex and *APOE*. While female sex confers higher risk of developing AD, *APOE4* interacts with sex to significantly impact AD pathways that involve neuroinflammation. Astrocytes and microglia support neuronal function and are key modulators of neuroinflammation. Our previous work has shown that microglia have protective interactions with plaques that are dependent on *APOE* genotype and sex [14]. Because astrocytes and microglia are intimately linked, we investigated the astrocyte interactions with amyloid plaques in relation to *APOE* status and biological sex.

## Methods

### Animals

The EFAD mice used in this study are hemizygous for 5xFAD transgenes and homozygous for targeted replacement of mouse *APOE* with human *APOE3* (E3FAD) or *APOE4* (E4FAD) [15]. Mice were euthanized at 6 months, an age characterized by significant AD-related neuropathology [16]. After mice were perfused with 4 °C PBS, the brains were extracted and fixed in 4% paraformaldehyde for 48 h. Four groups of mice were studied (*n* = 6 per group): male and female E3FAD, male and female E4FAD. This study was performed under an institutionally approved animal protocol and in accordance with the guidelines of the National Institutes of Health.

### Histochemistry

Fixed brains were sectioned (40 μm) in the sagittal plane with at least three medial, equidistantly spaced sections per brain stained using modifications of previously described protocols [14]. Staining batches were balanced across groups. In brief, sections were permeabilized in Triton X-100 for 15 min, followed by incubation at 4 °C with primary antibodies (diluted in blocking buffer) against glial fibrillary acid protein (GFAP, DAKO, 1:500), lysosomal-associated membrane protein 1 (LAMP1) (DHSB, 1:250), and or Ab (MOAB-2, Sigma-Aldrich, 1:100). After subsequent washing, sections were incubated with Alexa fluorophore-conjugated secondary antibodies (Invitrogen; anti-rabbit, anti-mouse, anti-rat) diluted 1:500 in blocking buffer. To label amyloidogenic plaques, immunostained sections were incubated with 0.5% THK-265 (THK; Sigma-Aldrich) for 20 min, washed with PBS, then mounted with ProLong Gold Antifade medium (Vectashield).

### Microscopy and image analyses

Image collection and analysis were performed as described [14] except as noted. In brief, a Zeiss-780 upright confocal microscope with ZEN imaging software (Zeiss) was used for image capture with researcher-blinded acquisition. Laser and detector settings were maintained across imaging sessions and high-resolution z-stack images were collected with optimal section depths (~ 0.35 µm). A 63× oil immersion objective (1.4 NA) was used to acquire regions of interest (ROI, 192.8 µm × 192.8 µm, 512 × 512 pixels, 16 bit) in the subiculum and cornu ammonis 1 (CA1) stratum radiatum fields of hippocampus, sampling areas with individual plaques. Analyses were performed using a custom ImageJ blinding plugin [17]. Images were de-noised and average projections were used for analysis.

Plaque coverage, size, and compaction were quantified from all plaques > 4 µm in diameter that were fully contained within the ROIs (≥ 3 per section, three sections per animal) and did not overlap with other plaques; 2–10 plaques were analyzed per animal. Plaque coverage was defined as the contact area between astrocyte processes and THK-265^+^ plaques (within 2 µm), calculated by summing arcs of plaque perimeters across three-dimensional stacks. Plaque area was manually determined in ImageJ and ranged from 10 to 108 µm^2^. Plaque circularity (a measure of compaction) was determined using the formula 4*π* × area/(perimeter)^2^.

Neuronal dystrophy was determined as a ratio, quantifying LAMP1 lysosomal staining density normalized to the corresponding THK-265^+^ plaque area. Analyses of individual astrocytes (3–41 cells/animal) included all non-overlapping GFAP-immunoreactive cells fully within the ROIs and within a 100 µm radius of THK-265^+^ plaques. Soma size was measured by manually identifying, outlining, and measuring GFAP-immunoreactive cell bodies using ImageJ. Astrocyte primary process number was manually determined as the number of processes emanating directly from GFAP-labeled somas as previously described [18].

### Statistical analyses

Two-way analysis of variance, with *APOE* genotype and sex as independent variables was performed using Prism (GraphPad Software, Inc. version 9), followed by Tukey *post-hoc* test to account for multiple comparisons. Kolmogorov–Smirnov and Shapiro–Wilk tests were used to test sample normality distribution. Kruskal–Wallis test was used to compare differences between groups whose distributions did not pass normality testing. Data are presented as box (mean and 25th and 75th quartiles) and whisker (minimum and maximum values) plots or as means (+ SEM). For all statistical tests, *p* values less than 0.05 were considered significant.

## Results

**Fig. 1:**
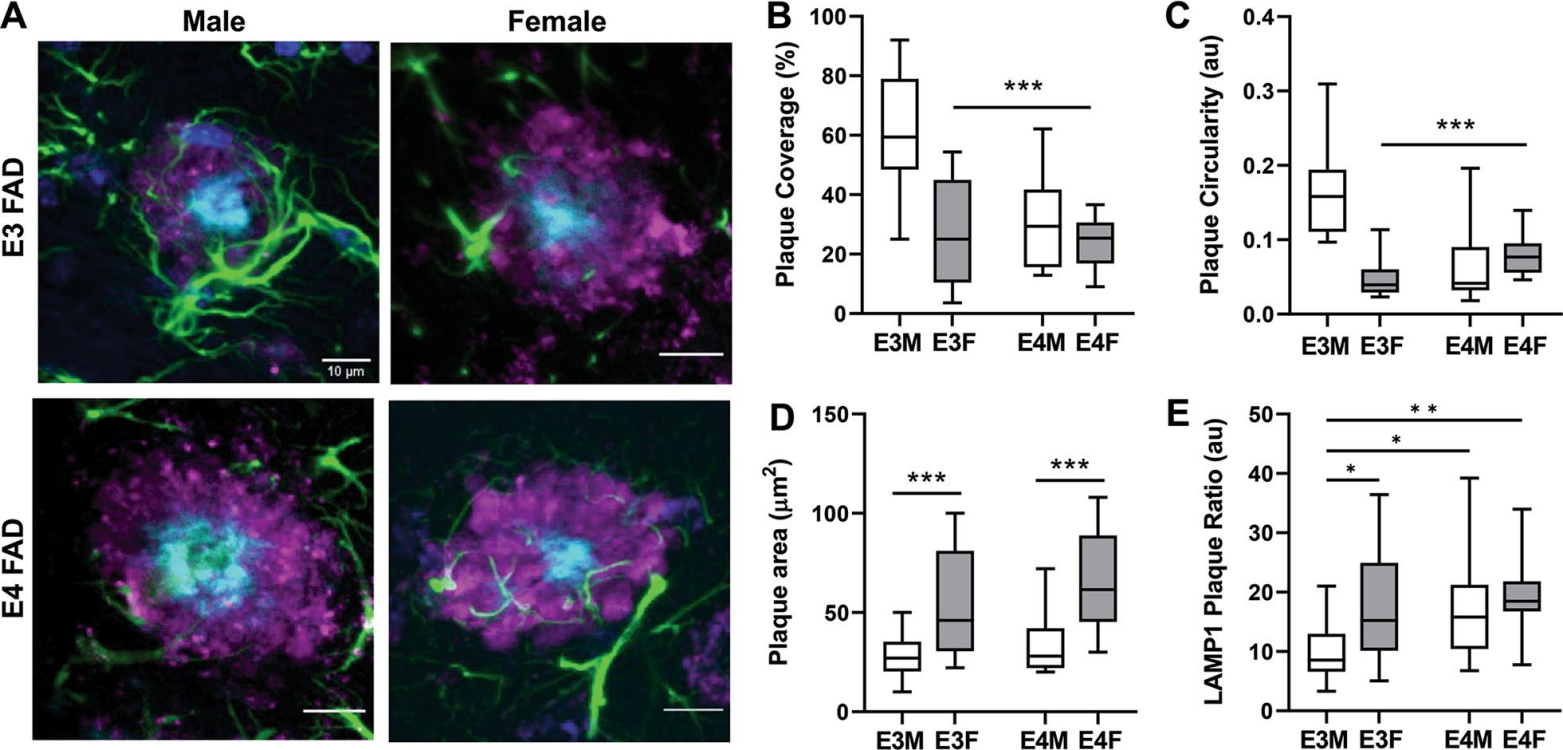
*APOE4* and female sex reduce protective astrocyte interactions with amyloid plaques. **A** Representative confocal images of THK-265-positive amyloid plaques (cyan), GFAP-immunoreactive astrocytes (green), LAMP1-positive dystrophic neurites (magenta) and cell nuclei labeled with 4′,6-diamidino-2-phenylindole (dark blue) in male and female E3FAD and E4FAD mice. Scale bars = 10 μm. Amyloid plaques were quantified for **B** coverage with GFAP-immunoreactive astrocyte processes, **C** plaque circularity, **D** plaque area, and **E** LAMP1-positive dystrophic neurites (normalized to plaque area) in male (E3M) and female (E3F) E3FAD and male (E4M) and female (E4F) EFAD mice. Data are presented as box (mean and 25th and 75th quartiles) and whisker (minimum and maximum values) plots from *n* = 6/group of mice at age 6 months. *p < 0.05, **p < 0.01, ***p < 0.001

To investigate astrocyte–plaque interactions and determine the effects of *APOE* genotype and biological sex, we first examined astrocyte association with Ab plaques. GFAP-labeled cells were colocalized predominantly with Ab-immunolabeled deposits that were also positive for the amyloid stain THK-265 in a manner that was similar across *APOE* genotype and sex (Additional file 1: Fig. S1). Next, we measured the close associations (within 2 µm) of GFAP-labeled processes with THK-265-labeled amyloid. The local interactions of these processes with plaques, termed plaque coverage, is consistent with prior descriptions of microglia creating a barrier-like net around amyloid deposits [9, 19]. Importantly, *APOE4* (*F*_(1,76)_ = 30.2, *p* < 0.0001) and female sex (*F*_(1,76)_ = 22.3, *p* < 0.0001) negatively affect the barrier-like interactions that astrocyte processes form around amyloid plaques, with a significant interaction between *APOE* and sex (*F*_(1,76)_ = 15.8, *p* < 0.0002) (Fig. 1A, B). Specifically, E3FAD male mice have the highest degree of astrocyte–plaque coverage, which is significantly lower in E4FAD male mice (*p* < 0.0001) and female E3FAD (*p* < 0.0001) and E4FAD (*p* < 0.0001) mice (Fig. 1A, B). Astrocyte–plaque coverage is not strictly related to astrocyte abundance as GFAP immunoreactive burden is lowest in male E3FAD and highest in female E4FAD mice (Additional file 1: Fig. S2), which parallels the increasing abundance of plaque pathology in EFAD mice with *APOE4* genotype and female sex [15, 20].

The plaque barrier that microglia form is associated with increased plaque compaction and reduced levels of dystrophic neurites (DNs) [9]. Plaque size and circularity were measured to understand how astroglial coverage affects plaque structure. There were significant main effects of sex (*F*_(1,72)_ = 18.7, *p* < 0.0001), *APOE* genotype (*F*_(1,72)_ = 8.9, *p* = 0.004), and *APOE* × sex interaction (*F*_(1,72)_ = 30.7, *p* < 0.0001) on plaque circularity with male E3FAD mice showing the highest values (Fig. 1C). Plaque area was significantly affected by sex (*F*_(1,72)_ = 37.4, *p* < 0.0001), such that female mice, regardless of *APOE* genotype, had significantly larger plaques (Fig. 1D). Plaque size differences do not appear to explain observed relationships as we noted numerous examples of strong astrocyte–plaque coverage with relatively large plaques in male E3FAD mice and weak astrocyte–plaque coverage with relatively small plaques in female E4FAD mice (Additional file 1: Fig. S3). We subsequently addressed plaque-associated LAMP1 labeling, a lysosomal marker known to exhibit high expression in plaque-associated DNs [21–24]. LAMP1 was used to establish whether astroglial plaque coverage was associated with neurite dystrophy. LAMP1 staining was significantly affected by sex (Fig. 1D, *F*_(1,72)_ = 7.4, *p* = 0.008) and *APOE* genotype (*F*_(1,72)_ = 8.3, *p* = 0.005). The highest levels of LAMP1 were observed in female E4FAD mice compared to male E3FAD mice (Fig. 1E, *p* = 0.003).

**Fig. 2:**
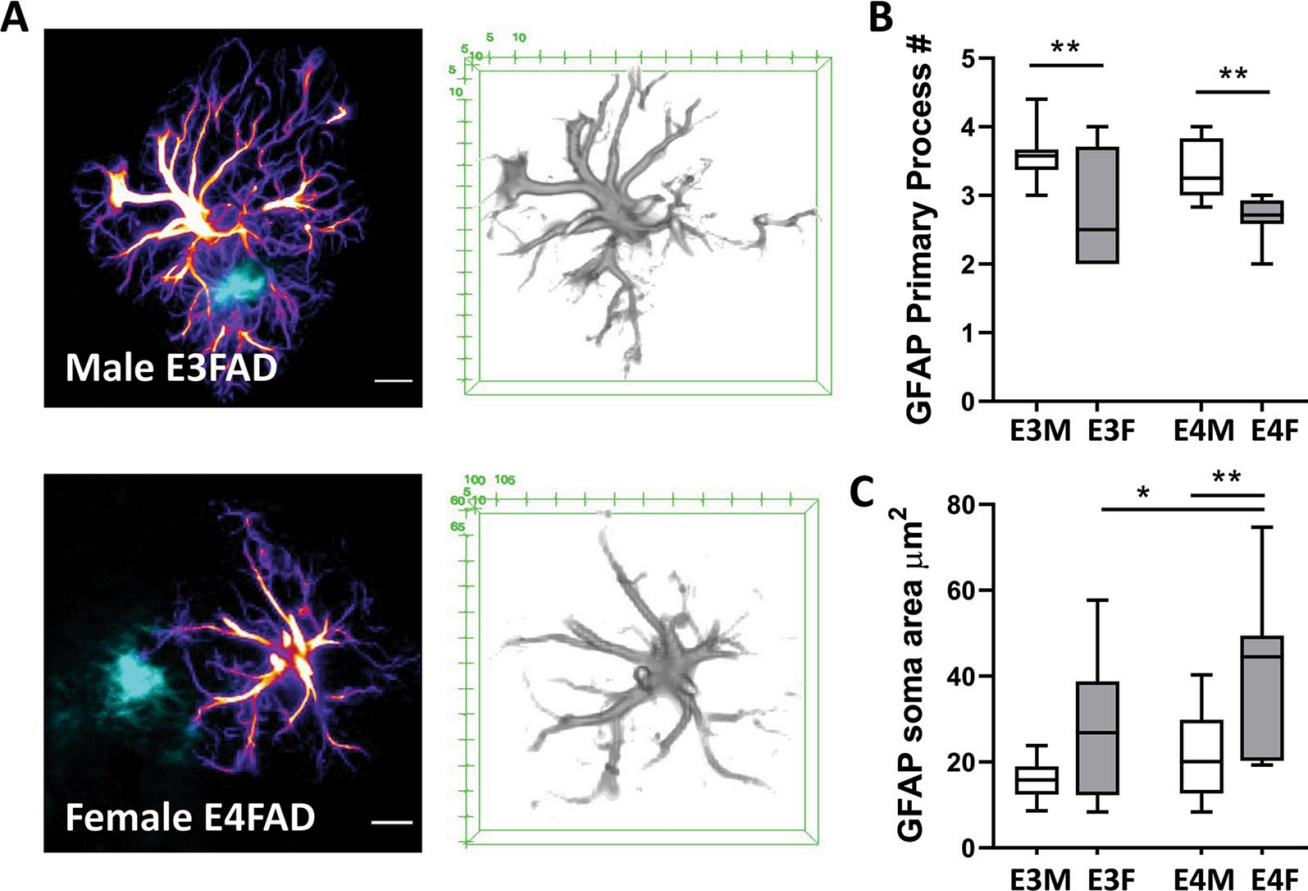
*APOE4* and female sex alter activation phenotype of near-plaque astrocytes. **A** Representative confocal images of THK-265-positive amyloid plaques (cyan) and individual GFAP-immunoreactive astrocytes (pseudo-colored orange–purple). Scale bars = 10 μm. Quantification of **B** astrocyte primary process number, and **C** astrocyte soma area in male (E3M) and female (E3F) E3FAD and male (E4M) and female (E4F) EFAD mice. Data are presented as box (mean and 25th and 75th quartiles) and whisker (minimum and maximum values) plots from *n* = 6/group of mice at age 6 months. *p < 0.05, **p < 0.01

Finally, the morphological phenotype of plaque-associated astrocytes was assessed to inform on activation state, which includes hypertrophy of astrocyte processes and increased soma size [18]. Significant main effects of both sex and *APOE* genotype on astrocyte soma area (sex: *F*_(1,52)_ = 21.6, *p* < 0.0001, *APOE*: *F*_(1,52)_ = 10.6, *p* = 0.002), and a significant main effect of sex on number of primary processes (sex: *F*_(1,45)_ = 26.9, *p* < 0.0001) were observed. Astrocytes from male E3FAD mice showed significantly higher process number and significantly lower soma area than female E4FAD mice (Fig. 2A–C, process number *p* = 0.0003, soma *p* < 0.0001). These results indicate that astrocytes proximal to amyloid plaques exhibit evidence of activation.

## Discussion

Astrocytes are important regulators of AD pathogenesis and are known to interact directly with amyloid plaques [25, 26]. However, the functional consequences of astroglial interactions with plaques have not been fully elucidated. This study describes novel astrocyte interactions with amyloid plaques in the EFAD mouse model of AD. We report astrocyte plaque coverage that is positively associated with plaque compaction and negatively associated with neuritic damage. These observations suggest that like microglia, astrocyte plaque interactions may serve a beneficial role by limiting neuronal associations with plaques.

Prior work has implicated astrocytes as regulators of AD pathogenesis [27]. However, it is still unclear how astrogliosis regulates disease progression given that activated astrocytes adopt phenotypes associated with both positive and negative outcomes [28]. The observed association between astrocyte–plaque coverage and both increased plaque circularity and reduced dystrophic neurites suggests that it is a beneficial function impaired in the context of *APOE4*. In line with this perspective, Mathur and colleagues observed that astrocyte interactions with plaque subtypes in AD brain were related to reduced cognitive impairment, including the finding of decreased astrocyte interactions with compact plaques predicting lower cognitive ability in a manner that was deleteriously affected by *APOE4* [29]. Conversely, since *APOE4* is associated with more rapid and extensive plaque pathology in EFAD mice [15], astrocyte–plaque interactions may reflect in part differing abundance of plaque pathology in age-matched *APOE3* versus *APOE4* mice, an issue that has been considered but remains unresolved [30]. In this case, one would predict that the patterns of astroglial–plaque interactions and Ab pathology show strong parallels across sex and *APOE* genotype, a relationship that is not found in our data. More specifically, the observed patterns of plaque coverage, plaque circularity and plaque-associated DNs show a clear separation between *APOE3* males and all other groups, which share similar outcomes (i.e., *APOE3* males > *APOE3* females, *APOE4* males, *APOE4* females). In contrast, Ab pathology in EFAD mice exhibits a different, graded pattern with *APOE3* males < *APOE3* females, *APOE4* males < *APOE4* females [20, 31]. The lack of concordance between astroglial–plaque interactions and measures of AD-related pathology suggest the possibility of inherent sex and *APOE* genotype effects. Indeed, there are numerous observations of sex × *APOE* interactions across multiple domains in the absence of significant Ab pathology in rodents and humans [32–36].

Our findings demonstrate that astrocyte coverage of plaques is significantly reduced by female sex. Specifically, we find that astrocytes in male E3FAD mice show greater astrocyte coverage, elevated plaque compaction, and lower levels of neurite injury; these effects are significantly diminished by female sex with both *APOE3* and *APOE4* genotypes. The mechanisms underlying observed sex differences in astrocyte actions remain to be elucidated. It is well-established that sex differences in AD are associated with both organizational effects of gonadal hormones during developmental periods [37, 38] and sex-specific abundance and age-related depletions of these hormones [39]. Astrocytes are known to participate in sexual differentiation of the brain and exhibit a range of sexual dimorphisms in function [40].

Our observations that astrocyte–plaque coverage is regulated by sex and *APOE* genotype parallels our prior observations with microglia [14]. More specifically, our findings indicated that microglia may protect neurons from amyloid by closely associating with amyloid plaques, but that *APOE4* and female sex yielded poorer microglial coverage. Astrocytes interact with fibrillar plaques in a complementary manner to that of microglia. It remains to be clarified whether astrocytes, microglia or perhaps their interactions are most important to glial plaque coverage. Recent proteomic work implicates changes in both astroglial and microglial protein markers as among the earliest events in AD development [41].

There are some limitations to this study. First, we labeled astrocytes with GFAP, a marker of astrocyte activation that is widely used but can be restrictive in revealing very fine astrocyte processes and is not a ubiquitous marker for all astrocytes [42]. Second, our findings are derived from a transgenic mouse model of AD, all of which have limitations that may restrict translation to human AD [43]. Third, this initial report did not vary parameters that can influence *APOE*-modulated glial outcomes in AD mice, including age [30] and brain region [44]. In addition, the analytic demands limited the total numbers of plaques and astrocytes studied, reinforcing the need for further study of the observed relationships.

In summary, these data provide the first clear evidence that astrocytes interact with amyloid plaques in a manner that is associated with both smaller plaque size and reduced plaque-associated neuritic damage. These astrocytic interactions are regulated by biological sex and *APOE* genotype. Collectively, these findings provide new insight into the role of glia as contributors to the relationships among sex, *APOE*, and AD.

## Supplementary Information

**Additional file 1: Figure S1.** Astrocytes colocalize with amyloid plaques across *APOE* genotypes and sex. **Figure S2.** Astrocyte immunoreactive load varies across *APOE* genotypes and sex. **Figure S3.** Astrocyte immunoreactive load varies across *APOE* genotypes and sex.

## Data Availability

The data sets used and/or analyzed during the current study are available from the corresponding author on reasonable request.

## Abbreviations

AD: Alzheimer’s disease
Aβ: β-Amyloid
*APOE*: Apolipoprotein E
CA1: Cornu ammonis 1
CNS: Central nervous system
DNs: Dystrophic neurites
GFAP: Glial Fibrillary Acid Protein
LAMP1: Lysosomal-associated membrane protein 1
ROI: Region of interest
