# Supplementary material for: APOE genotype and biological sex regulate astroglial interactions with amyloid plaques in Alzheimer’s disease mice
Source: J Neuroinflammation. 2022 Dec 1;19:286. doi: 10.1186/s12974-022-02650-4 (PMC9714101; doi:10.1186/s12974-022-02650-4)
Supplement: Supplementary file 1 — Additional file 1: Figure S1. Astrocytes colocalize with amyloid plaques across APOE genotypes and sex. Figure S2. Astrocyte immunoreactive load varies across APOE genotypes and sex. Figure S3. Astrocyte immunoreactive load varies across APOE genotypes and sex. [file 12974_2022_2650_MOESM1_ESM.docx]

**Additional file 1**

**
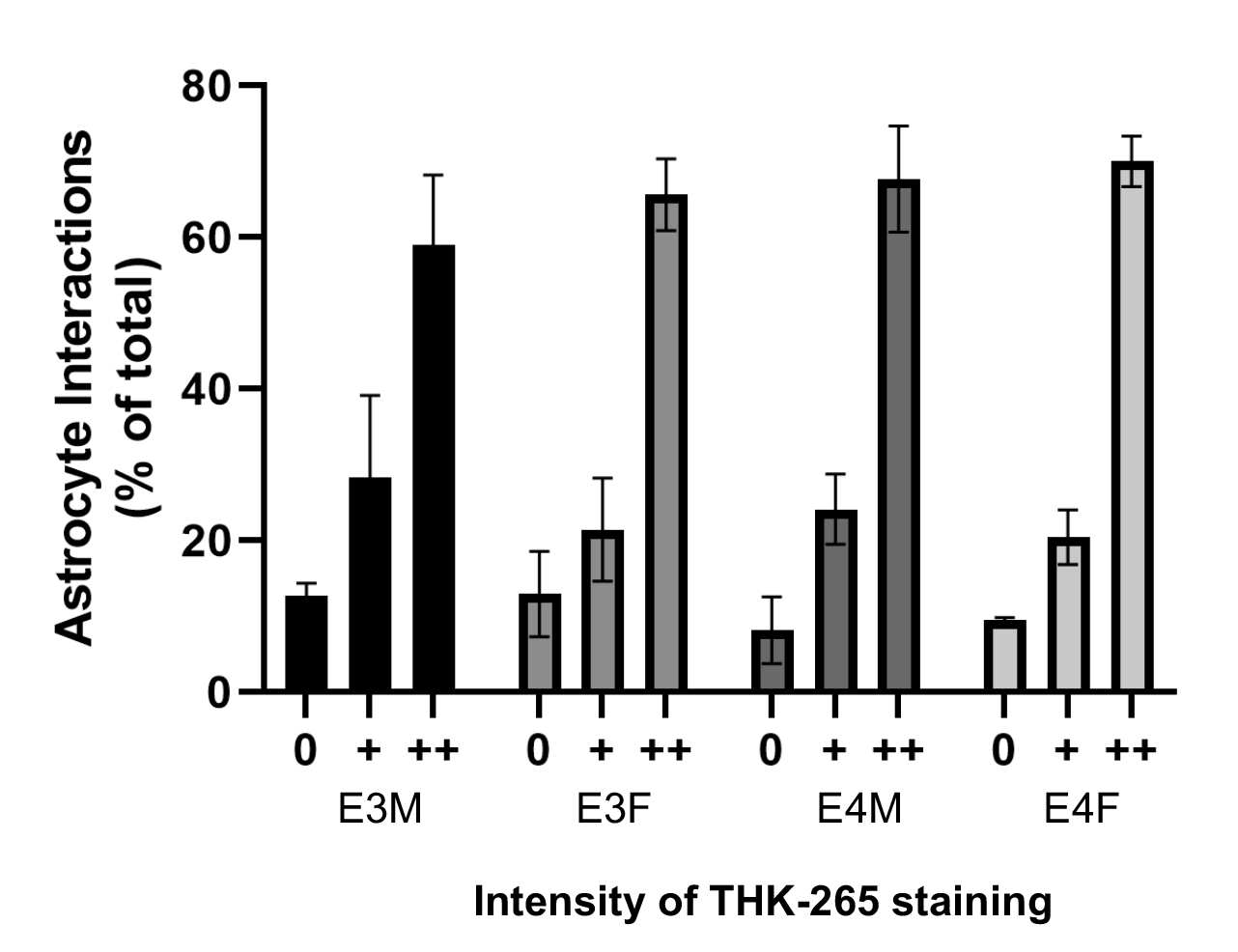
**

**Figure S1. Astrocytes colocalize with amyloid plaques across *APOE* genotypes and sex.** Plaques from EFAD mice (n=3 per group; age 6 months) were immunolabeled for Aβ (MOAB-2) and astrocytes (GFAP), then stained with the amyloid dye THK-265. Aβ plaques (n=20 per brain) were scored for (i) the presence or absence of colocalized GFAP-labeled cells or processes, and (ii) the intensity of THK-265 labeling: absent (0), positive but weak (+), or strongly positive (++). Data show percentage (+SEM) of total plaques with colocalized astrocyte stratified according to THK-265 staining intensity in male (E3M) and female (E3F) E3FAD and male (E4M) and female (E4F) E4FAD mice. There was a main effect of THK-265 intensity (*F*(_2,30_) = 105.4, p < 0.0001) but not *APOE* genotype (*F*(_1,30_) = 0.1, p = 0.99) and no interaction (*F*(_2,30_) = 0.9, p = 0.42) by two-way ANOVA.

**
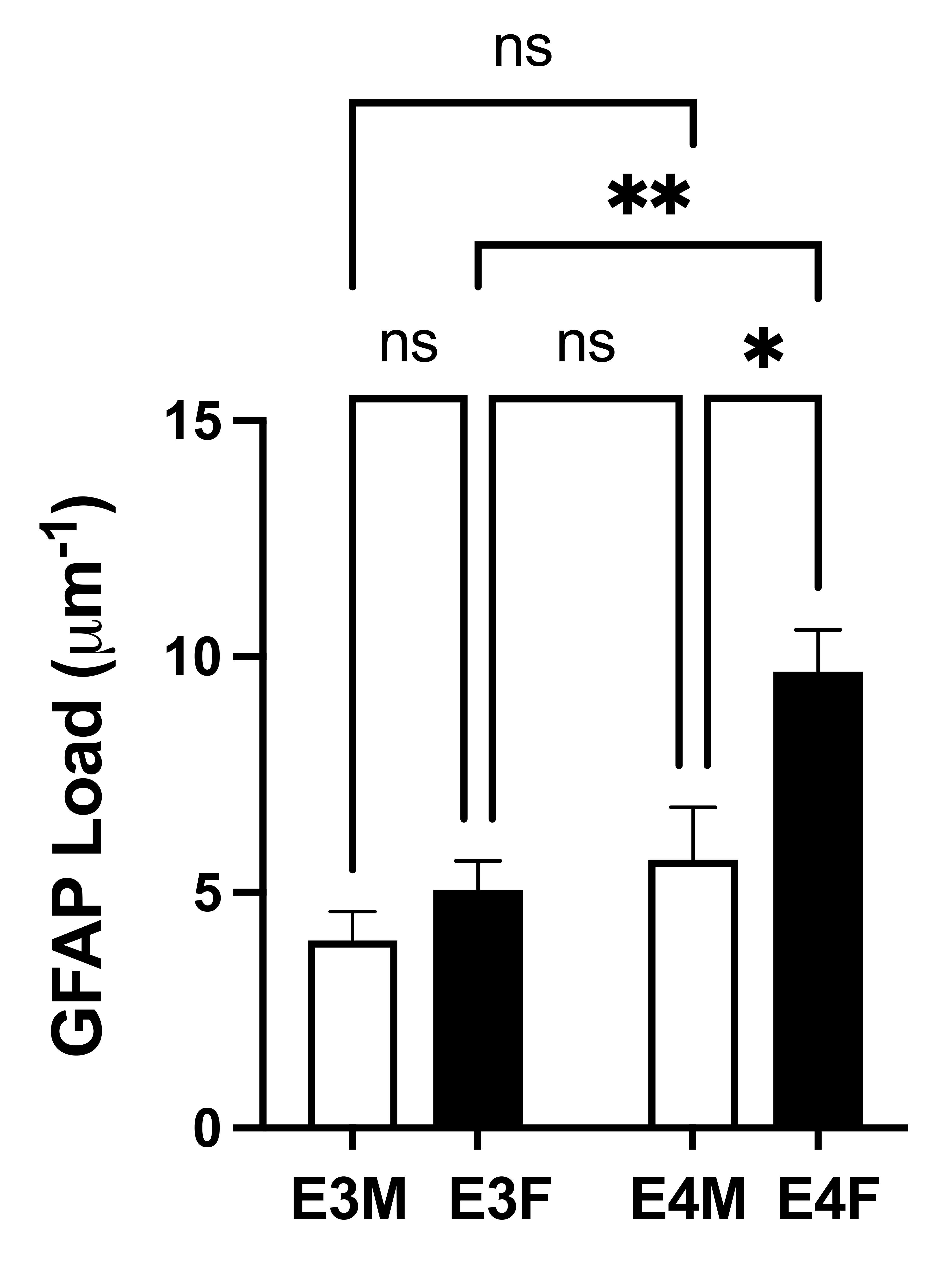
**

**Figure S2. Astrocyte immunoreactive load varies across *APOE* genotypes and sex.** Confocal images of GFAP immunolabeling were converted to 8-bit grayscale and thresholded at a constant value using NIH ImageJ software to yield binary images of positive and negative labeling, as previously described (Stephen *et al.*, 2019). The immunoreactive load was calculated as the percentage of pixels within the entire field with positive labeling. Data show mean levels (+SEM) of GFAP immunoreactive load in male (E3M) and female (E3F) E3FAD and male (E4M) and female (E4F) EFAD mice (n=6/group, age 6 months). There were significant main effects of *APOE* genotype (*F*(_1,34_) = 8.4, p = 0.006) and sex (*F*(_1,34_) = 13.2, p = 0.0009) and no interaction (*F*(_1,34_) = 1.3, p = 0.1) by two-way ANOVA. * p < 0.05, ** p < 0.01, ns: not significant.

**
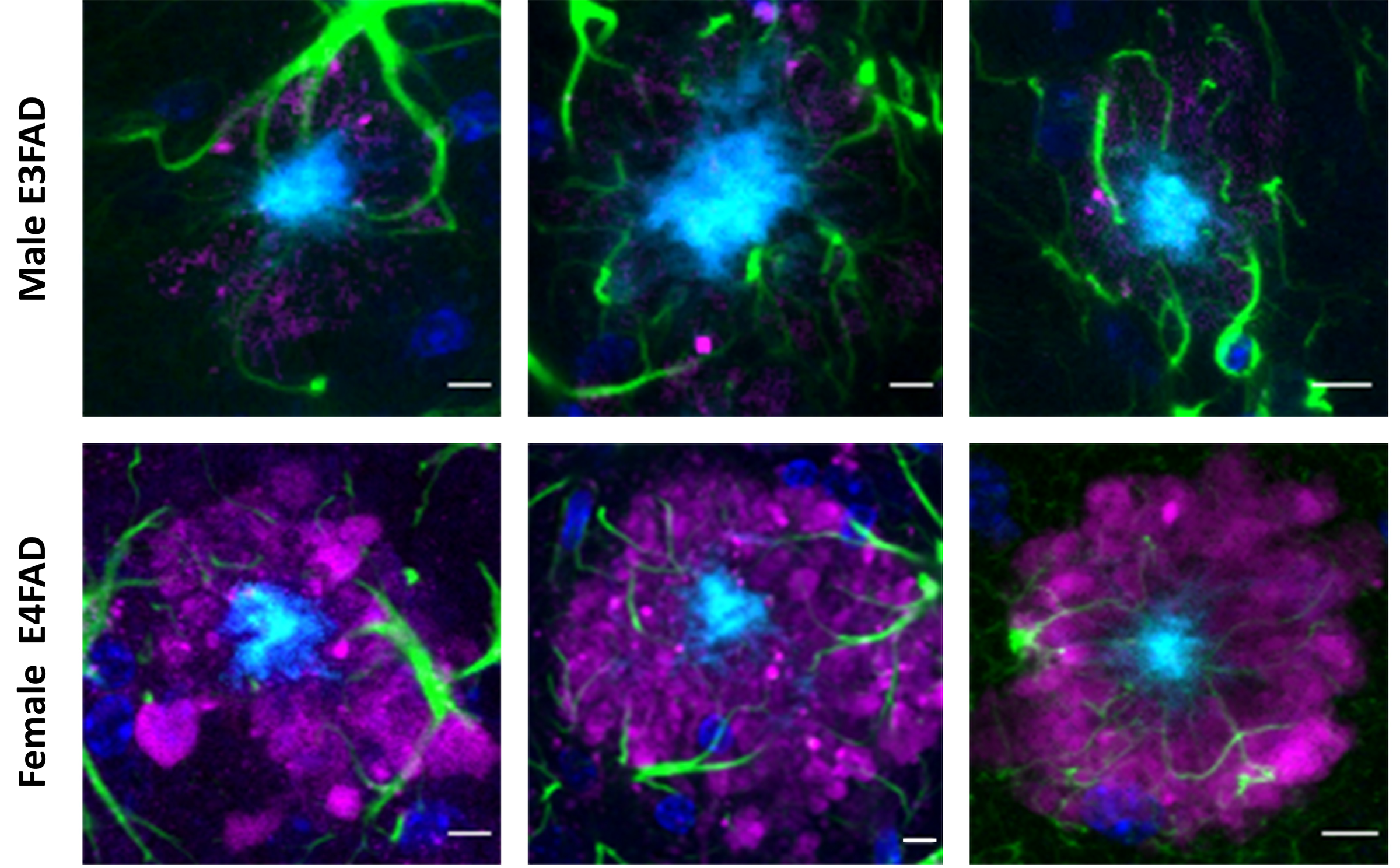
**

**Figure S3. Astrocyte immunoreactive load varies across *APOE* genotypes and sex.** Confocal images of THK-265-positive amyloid plaques (cyan), GFAP-immunoreactive astrocytes (green), LAMP1-positive dystrophic neurites (magenta) and cell nuclei labeled with 4’,6-diamidino-2-phenylindole (dark blue) in three different male E3FAD and female E4FAD mice (age 6 months). Scale bars = 5 μm.
